# Hygienic practices and diarrheal illness among persons living in at-risk settings in Kabul, Afghanistan: a cross-sectional study

**DOI:** 10.1186/s12879-016-1789-3

**Published:** 2016-08-31

**Authors:** Mohammad Yousuf Mubarak, Abram L. Wagner, Mari Asami, Bradley F. Carlson, Matthew L. Boulton

**Affiliations:** 1 Department of Microbiology, Kabul Medical University, Jamal Mina, 3rd District, University Road, Kabul, Afghanistan; 2 Department of Epidemiology, University of Michigan, 1415 Washington Heights, Ann Arbor, MI USA; 3 Area on Water Management, Department of Environmental Health, National Institute of Public Health, 2-3-6 Minami, Wako, Saitama 351-0197 Japan

**Keywords:** Drinking water, Hand washing, Sanitation, Hygiene, Diarrheal diseases

## Abstract

**Background:**

Sustained civil and military conflict, resulting in large numbers of internally displaced persons (IDP), in combination with rapid urbanization has strained public health and sanitation within cities in Afghanistan. In order to examine the association between preventive sanitary behaviors and diarrhea within two high risk settings located within Kabul, Afghanistan, this study aimed to evaluate the prevalence of hygienic practices and diarrheal illness in an IDP camp and an urban slum.

**Methods:**

In this cross sectional study, a convenience sample of residents of an IDP camp and an urban slum in Kabul, Afghanistan, was used. Participants were asked to describe their hygienic practices and interviewers independently documented household sanitation. The knowledge and attitudes about and practice of hygienic activities to prevent diarrhea were compared between the two settings.

**Results:**

Two hundred participants, 100 from each setting, were enrolled. Knowledge, attitudes, and practices regarding hygienic activities to prevent diarrhea were greater among the slum dwellers than the IDP. Fewer than half of participants washed their hands with soap before eating or after eating: 31 % of slum dwellers washed before eating compared to 11 % of IDPs (*P* = 0.0050), and 25 % of slum dwellers washed after defecating compared to 4 % of IDPs (*P* = 0.0020). The IDPs were more likely to share a latrine (*P* = 0.0144) and less likely to disinfect their latrine than slum dwellers. Diarrhea in the household within the past 3 months was more common in the IDP camp (54 %) than the slum (20 %) (*P* = 0.0020).

**Conclusions:**

Even though certain sanitary and hygienic practices were more common among slum dwellers than IDPs, the lack of hygienic activities in both setting indicates that interventions to change behavior, like increasing the availability of soap and encouraging hand washing, are needed. Any initiative will have to be developed in the context of pervasive illiteracy among persons in both of these settings.

**Electronic supplementary material:**

The online version of this article (doi:10.1186/s12879-016-1789-3) contains supplementary material, which is available to authorized users.

## Background

Major challenges have confronted efforts to improve public health in Afghanistan over the last several decades due to sustained military and civil conflict. The Soviet invasion in 1979, the Taliban take-over in the late 1990s, and U.S. military activities starting in 2001 have limited the development of a stable government which could provide its population with basic health services [1]. In recent years, Afghanistan has had to support a large number of internally displaced persons (IDP), that is, people who “have not crossed an international border to find sanctuary but have remained inside their home countries [because of] armed conflict, generalized violence, [or] human rights violations” [2]. Displacement in Afghanistan has resulted, in part, from armed conflict between the Afghan army and non-state armed groups—including the Taliban, inter-tribal disputes, and natural disasters [3, 4]. Data suggest that the number of IDPs in Afghanistan has increased in recent years, from 400,000 in 2012 to 600,000 in 2014 [5], and 60 % of IDPs in Afghanistan are children [6]. Consequently, the plight and health status of IDPs in Afghanistan is of special and growing concern.

The influx of IDPs, economic migrants from rural areas, and returning refugees from Pakistan and Iran into Afghanistan, has stressed the infrastructure of cities [7]. Afghanistan’s rate of urbanization (the projected change in the size of the urban population), approaches 6 % and is one of the highest in the world [8]. As a result, nearly 75 % of the urban population, or five million persons, are living in slums and other underdeveloped areas [9], which lack access to governmental municipal services including delivery of potable, piped water and waste disposal via municipal sewers. The conditions in these underdeveloped areas varies, but range widely from camps where IDPs live in tents and other temporary housing to slums or shantytowns which are not connected to municipal infrastructure with inhabitants live in inadequately constructed, albeit semi-permanent, housing. Both IDP camps and urban slums represent settlements that tend to be characterized by greater environmental risks that predispose residents to poorer health outcomes like diarrheal illness [10, 11].

The health and socioeconomic status of Afghans is quite low compared to other developing nations; Afghanistan ranks 171 out of 188 countries in terms of its Human Development Index (HDI), which at 0.465 is the lowest of any country outside of Africa [12]. The proportion of the population considered living at or below poverty line is 36.5 %, and only 31.4 % of adolescents and adults aged 15–24 are literate [13]. Infant mortality in Afghanistan is high with a rate of 71 per 1000 live births compared to 15 in Iran and 24 in Kyrgyzstan, two neighboring countries [14]. Little research has been conducted in Afghanistan on most public health issues, including the use of hygienic practices to prevent diarrhea, despite the finding of one survey in which 26.4 % of children had diarrhea in the preceding 2 weeks [13]. The lack of public health research on diarrheal illness and other public health problems is characteristic of persons and families who are living in IDP camps and in slums. In this study, we conducted surveys in two high risk settings for diarrheal illness in Kabul, Afghanistan; an IDP camp and an urban slum, in order to assess the association between use of hygienic practices and the prevalence of diarrhea in the household.

## Methods

### Study population

This cross-sectional study was conducted in Kabul, Afghanistan in August and September, 2012. The two settings selected for sampling were Charahi Qambar, an IDP camp, and Company Sare Karez, an urban slum both of which are both located in Kabul, Afghanistan. Charahi Qambar consists of approximately 874 families living mostly in tents, that house about 6900 individuals who are largely residents originally from Helmand Province in southern Afghanistan who were mostly dislocated by war. The urban slum, Company Sare Karez, is composed of approximately 1050 households (7000 to 9000 individuals) living in poorly constructed mud homes which lack indoor plumbing for drinking water or sewage. Because the study took place during Afghanistan’s summer season, the hot weather exacerbated water shortages and the poor sanitary conditions [15].

Within both the IDP camp and the slum, 100 households were chosen. The IDP camp households were systematically sampled from a list of all households provided by the unofficial head of the camp. This leader had been informally selected by the camp dwellers to serve as a liaison between the camp and outside agencies, including the Afghan government and foreign non-governmental organizations (NGOs). The list of households was revised approximately every 3 months to document people who had either left or entered the camp in order to provide the government and NGOs with a list of people needing support. No comparable household list was available for the slum, and so a convenience sample was used. The interviewers started at a random point within the slum, and selected every third to fifth household thereafter. The interval between households sampled differed because interviewers tried to purposively sample a diversity of households, for instance choosing to go to a new block or to a household with different exterior characteristics from the previous enrolled household. In both locations, study interviewers went to the selected houses and asked to speak with either the adult female or male head of household, who were then interviewed for the survey. If the head of household was not available, two more attempts were made to visit the house at different days and at different times. If the head of camp or area representative had the household’s phone number, then the potential participant was called to inform him or her about the study and to set up a time for the interviewer to conduct the interview. If a selected household declined to participate, the next neighboring household was approached about participating in the study.

### Variables

The questionnaire was available in both Dari and Pashto, the two main languages of Kabul. Participants were asked about personal sociodemographic characteristics and their knowledge, attitudes, and practice (KAP) of hygienic measures associated with prevention of diarrhea, including handwashing, use of drinking water, and appropriate waste disposal. Sociodemographic variables include sex, age, household size, number of children in house, literacy, and length of time living in current location. Handwashing KAP was assessed by asking respondents about hand-washing, washing before eating and after defecating, and access to a sink, i.e., a washbin next to the latrine (no household in the study had running water). Questions on drinking water asked about source, time required to access, storage, and whether participants treated water or boiled prior to use. Characteristics of latrine use included questions addressing sharing of latrines between households, latrine type (vault, open pit, pour-flush), location, and whether it was disinfected. Interviewers directly documented the time required to reach water and the distance between the latrine and drinking water source.

Participants were asked about any episodes of diarrhea in the household in the past 3 months; information on the number of individuals with diarrhea in the household was not collected. If yes, they were asked specifically about diarrheal characteristics (e.g. presence of mucus, blood, and/or worms in the diarrhea) and use of health care practices to treat diarrhea including administration of oral rehydration solution (ORS) and whether a doctor was consulted. The recall period of 3 months was chosen because the study was conducted at the end of summer, and we wished to estimate the occurrence of disease in the entire season. This is the hot season in Afghanistan, and in similar locations, for example Vellore, India [16], this is the season with highest incidence of community diarrhea.

The questionnaire was developed based on those used in the Johns Hopkins Safe Water System Project Report from Afghanistan [17]. The questionnaire was pilot tested prior to study launch. Interviewers were medical students and trained staff with prior medical background. The questionnaire in Dari and English is available in the Additional file 1.

### Statistical analysis

The distribution of sociodemographic characteristics and KAP variables was compared between IDPs and slum dwellers. Differences were assessed using Pearson’s chi-square test or Fisher’s exact test (if cell count <5). Bivariate *p*-values were corrected for multiple testing through the sequential Holm-Bonferroni Method. In the multivariable logistic model, the occurrence of diarrhea in the household was regressed onto each sociodemographic characteristic and KAP variable. Each model adjusted for the location (IDP camp and slum) because this was a putative confounder, but was otherwise unadjusted.

For all analyses, a *P*-value of <0.05 was considered statistically significant. Analyses were performed in SPSS version 16.0 (IBM Corporation, Armonk, NY, USA) and SAS version 9.3 (SAS Institute, Cary NC, USA). The Holm-Bonferroni-corrected *P*-values were computed in R version 3.0.3 (R Foundation for Statistical Computing, Vienna, Austria).

## Results

### Sociodemographic characteristics

In order to obtain a final sample size of 200 persons consisting of 100 from an IDP camp and 100 from a slum, 201 individuals were contacted. One individual in the IDP camp refused to participate in the study, and a neighboring household was enrolled in order to attain a sample size of 100 in each of the two sites. Slightly more respondents were males than females (52 % male in IDP camp, and 64 % in slum), and most were between the ages of 38 and 51 (60 % in IDP camp and 51 % in slum). Average household size was larger in the slum than IDP camp: 76 % of households had ≥8 individuals in the slum, compared to 55 % in the IDP camp (*P* = 0.0144). Literacy among respondents was higher in the slum (60 %) than in the IDP camp (38 %) (*P* = 0.0144), and participants and their household generally relocated to the IDP camp more recently (63 % in past year) compared to the participants in slum (22 %) (*P* = 0.0020) (see Table 1).

**Table 1:** Sociodemographic characteristics of respondents in Kabul, Afghanistan, 2012

	IDP camp	Slum	*P*-value^a^
Sex			
Male	52 (52 %)	64 (64 %)	0.3084
Female	48 (48 %)	36 (36 %)	
Age			
23–37	24 (24 %)	14 (14 %)	0.0550
38–43	33 (33 %)	24 (24 %)	
44–51	27 (27 %)	27 (27 %)	
52 and older	16 (16 %)	35 (35 %)	
Household sizeAge			
< 8	45 (45 %)	24 (24 %)	0.0144*
8 or more	55 (55 %)	76 (76 %)	
Children in house			
1	21 (21 %)	18 (18 %)	1.0000
2	34 (34 %)	33 (33 %)	
3 or more	44 (44 %)	49 (49 %)	
Literacy			
Literate	38 (38 %)	60 (60 %)	0.0144*
Illiterate	62 (62 %)	40 (40 %)	
Residency length			
< 1 year	63 (63 %)	22 (22 %)	0.0020*
1 or more years	37 (37 %)	78 (78 %)	

Notes

*IDP* internally-displaced person

*P < 0.05

^a^Adjusted for multiple testing (for statistical tests in Tables 1, 2, and 3 together) with the Holm-Bonferroni method

### Knowledge, attitudes, and practices on diarrhea prevention

Table 2 shows handwashing behaviors and attitudes towards hygienic measures for the camp and the slum. Fewer participants in the IDP camp (64 %) thought washing dirty hands was necessary as compared to participants in in the slum (84 %) (*P* = 0.0117), although this may have been based, at least in part, on a lack of available water for some. Respondents from the slum were also more likely to wash hands with soap before eating (*P* = 0.0050) and after defecating (*P* = 0.0020), compared to those in the IDP camp, although fewer than half did so in either setting.

**Table 2:** Hygienic practices of study population in Kabul, Afghanistan, 2012

	IDP camp	Slum	P-value^a^
Is it necessary to wash dirty hands?			
No	36 (36 %)	16 (16 %)	0.0117*
Yes	64 (64 %)	84 (84 %)	
Why is it not necessary to wash dirty hands?^b^			
Water unavailable	17 (47 %)	10 (63 %)	
Soap unavailable	2 (6 %)	1 (6 %)	
Not necessary	10 (28 %)	2 (13 %)	
Don't know	7 (19 %)	3 (19 %)	
Do you wash hands with soap before eating?			
No	89 (89 %)	69 (69 %)	0.0050*
Yes	11 (11 %)	31 (31 %)	
Do you wash hands with soap after defecating?			
No	96 (96 %)	75 (75 %)	0.0020*
Yes	4 (4 %)	25 (25 %)	
Presence of sink			
No	61 (61 %)	24 (24 %)	0.0020*
Yes	39 (39 %)	76 (76 %)	
Drinking water source			
Unprotected	91 (91 %)	43 (43 %)	
Traditional Karez^c^	0 (0 %)	41 (41 %)	
Protected	9 (9 %)	16 (16 %)	
Time to get drinking water			
> 10 min	50 (51 %)	51 (51 %)	1.0000
10 min or less	49 (49 %)	49 (49 %)	
Drinking water stored in separate container			
No	59 (59 %)	22 (22 %)	0.0020*
In uncovered container	12 (12 %)	10 (10 %)	
In covered container	29 (29 %)	68 (68 %)	
Duration of time storing drinking water			
More than 1 day	47 (47 %)	21 (21 %)	0.0020*
1 day	53 (53 %)	79 (79 %)	
Do you treat water before drinking?			
No	84 (84 %)	47 (47 %)	0.0020*
Yes	16 (16 %)	53 (53 %)	
Method of treating water^d^			
Boiling	13 (59 %)	48 (79 %)	
Bleach	5 (23 %)	8 (13 %)	
Cloth filter	3 (14 %)	4 (7 %)	
Solar disinfection	1 (5 %)	1 (2 %)	
Is it necessary to boil water every time before drinking?			
No	53 (53 %)	24 (24 %)	0.0020*
Yes	47 (47 %)	76 (76 %)	
Is running water safe to drink?			
No	58 (58 %)	70 (70 %)	0.3084
Yes	42 (42 %)	30 (30 %)	
Latrine shared between households			
No	58 (58 %)	78 (78 %)	0.0144*
Yes	42 (42 %)	22 (22 %)	
Type of latrine			
Vault	40 (40 %)	35 (36 %)	
Elevated Vault	43 (43 %)	32 (33 %)	
Pit	0 (0 %)	16 (16 %)	
Pour-flush	0 (0 %)	7 (7 %)	
Other	0 (0 %)	3 (3 %)	
None	17 (17 %)	4 (4 %)	
Latrine location^e^			
Outside	50 (60 %)	48 (50 %)	
Inside	33 (40 %)	48 (50 %)	
Method of disinfecting latrine			
Ash	30 (36 %)	71 (74 %)	
Salt	0 (0 %)	6 (6 %)	
Chlorine	0 (0 %)	4 (4 %)	
Do not disinfect	53 (64 %)	15 (16 %)	
Distance between latrine and drinking water source^e^			
More than 10 m	13 (16 %)	48 (50 %)	
10 m or less	70 (84 %)	48 (50 %)	

Notes

*IDP* internally-displaced person

*P < 0.05

^a^Adjusted for multiple testing (for statistical tests in Tables 1, 2, and 3 together) with the Holm-Bonferroni method

^b^Among respondents who said washing dirty hands was unnecessary

^c^A Karez is a traditional system to channel water from aquifers under hills or mountains into a settlement

^d^Among respondents who treat water. Respondents could select multiple methods

^e^Among respondents with a latrine

Drinking water KAPs differed across the two settings, with participants from the slum engaging in more sanitary practices than those in the IDP camp. For example, the slum dwellers were more likely to store water in a covered container (68 %) compared to those in the IDP camp (29 %) (*P* = 0.0020), and they stored water for shorter periods of time (53 % for ≤1 day at most, compared to 79 % of people in IDP camps who stored water for ≤1 day, *P* = 0.0020), and slightly more than half (54 %) treated their water, compared to 6 % in the IDP camp (*P* = 0.0020). In both the slum and the camp boiling was the most common form of treating water, and time between the household and the drinking water source was comparable for both groups with approximately half requiring over 10 min to reach the water source.

Households did not typically share a latrine with another household, but the proportion who did not share (70 %) was higher in the slums than in the IDP camp (58 %) (*P* = 0.0144). In camps and slums, the two most common types of household latrines were vault and elevated vault, and latrines were about as likely to be located outside the household as inside. A large majority (84 %) of slum households disinfected their latrine whereas only 36 % of those in the IDP camp did so, with wood ash the preferred disinfection agent.

### Diarrhea characteristics

Table 3 shows the diarrheal occurrence and diarrheal symptoms, and prevention measures undertaken by study participants. Report of any household diarrhea within the past 3 months was more common in the IDP camp (54 %) than the slum (20 %) (*P* = 0.0020). Among those households which reported diarrhea, symptomology differed between the two locations. Worms in the diarrheal stool occurred in 76 % of IDP households and 60 % of slum households; blood occurred in 28 % of IDP household with diarrhea but only 10 % of slum households with diarrhea; and mucus was seen in 41 % of the diarrhea from the IDP camp but only 10 % of the diarrhea from the slum household. Fewer than half of participant households (47 %) in the IDP camp consulted a doctor during an episode of diarrhea, compared to three-quarters of the slum (*P* = 0.0033). Reasons for not consulting a doctor included lack of resources to do so (41 % of IDP camp, 40 % of slum) and absence of an available doctor available (44 % of IDP camp, 60 % of slum).

**Table 3:** Diarrheal illness and associated knowledge in study population, Kabul, Afghanistan, 2012

	IDP camp	Slum	P-value^a^
Diarrhea in household in past 3 months			
Yes	54 (54 %)	20 (20 %)	0.0020*
No	29 (29 %)	80 (80 %)	
Not sure	17 (17 %)		
Diarrhea characteristics			
Mucus in diarrhea	22 (41 %)	2 (10 %)	
Blood in diarrhea	15 (28 %)	2 (10 %)	
Worms in diarrhea	41 (76 %)	12 (60 %)	
Normal stool	13 (24 %)	13 (65 %)	
Give ORS to child with diarrhea^b^			
Yes	27 (50 %)	17 (85 %)	
No	16 (16 %)	0 (0 %)	
Not sure	11 (11 %)	3 (3 %)	
Consult doctor during episode of diarrhea			
Yes	47 (47 %)	75 (75 %)	0.0033*
No	41 (41 %)	20 (20 %)	
Not sure	12 (12 %)	5 (5 %)	
Reasons for not consulting doctor^c^			
No access	18 (44 %)	12 (60 %)	
Do not have resources	17 (41 %)	8 (40 %)	
Other	6 (15 %)	0 (0 %)	
Knowledge that diarrhea can be fatal			
Yes	32 (32 %)	67 (67 %)	0.0020*
No	28 (28 %)	17 (17 %)	
Not sure	40 (40 %)	16 (16 %)	

Notes

*IDP* internally-displaced person

*P < 0.05

^a^Adjusted for multiple testing (for statistical tests in Tables 1, 2, and 3 together) with the Holm-Bonferroni method

^b^Among respondents who answered that there was an episode of diarrhea in household

^c^Among respondents who do not consult doctor during episode of diarrhea

Nineteen multivariable logistic models separately regressed the occurrence of diarrhea in the household onto sociodemographic characteristics and KAP variables (Table 4). Participants who responded that they did not have diarrhea in the household or did not know if there was diarrhea in the household were grouped together. In each model, which only included the respective risk factor as well as the location (IDP camp and slum), only location was significant. Participants living in the IDP camps had 4.70 times higher odds of diarrhea than slum dwellers (95 % CI: 2.51, 8.80).

**Table 4:** Associations between hygienic practices and diarrhea, Kabul, Afghanistan, 2012

	OR^a^	95 % Confidence Interval	
Sex			
Male vs female	0.75	0.40	1.39
Age			
23–37	1.12	0.43	2.88
38–43	0.84	0.35	2.01
44–51	0.96	0.40	2.31
52 and older	ref		
Household size			
< 8 vs 8 or more	1.53	0.80	2.90
Children in house			
1	0.97	0.42	2.23
2	1.35	0.68	2.71
3 or more	ref		
Literacy			
Illiterate vs literate	0.70	0.37	1.31
Residency length			
1 or more years vs <1 year	0.58	0.29	1.18
Need to wash dirty hands?			
No vs yes	0.54	0.26	1.12
Wash hands with soap before eating?			
No vs yes	2.13	0.88	5.13
Wash hands with soap after defecating?			
No vs yes	0.81	0.31	2.14
Presence of sink			
No vs yes	0.73	0.37	1.43
Time to get drinking water			
>10 min vs 10 min or less	0.95	0.52	1.77
Drinking water stored in separate container			
No vs yes	0.54	0.26	1.14
In uncovered container	0.64	0.22	1.88
In covered container	ref		
Duration of time storing drinking water			
> 1 day vs 1 day	1.63	0.85	3.13
Treat water?			
No vs yes	0.79	0.38	1.63
Need to boil water before drinking?			
No vs yes	0.68	0.35	1.33
Is running water safe to drink?			
No vs yes	0.78	0.42	1.48
Latrine shared between households			
No vs yes	1.61	0.82	3.19
Do you have a latrine?			
No vs yes	1.28	0.49	3.36
Setting^b^			
IDP camp vs slum	4.70	2.51	8.80

Notes

^a^Models were adjusted only for two independent variables: the factor of interest (statistics presented) and setting (IDP camp vs slum)

^b^This model only included the one independent variable

## Discussion

In this cross-sectional study of hygienic practices and diarrheal illness conducted in two rarely examined, high-risk settings in Kabul, Afghanistan, we found understanding and use of sanitation was low and the prevalence of diarrhea within households was high among both slum dwellers and internally displaced persons living in camps. Moreover, the diarrheal presentation was often severe, marked by blood or mucus, which could be indicative of shigellosis or infection with Shiga toxin-producing *Escherichia coli* [18]. Although participant households in the slums were more likely to regularly engage in preventive behaviors than were participants in the camps, including hand washing before eating and after defecating and treating drinking water before use, there remain clear opportunities for individual- and systems-level interventions that encourage implementation of better sanitation at the individual, household, and community levels.

One previous study in Afghanistan also found high levels of recent diarrhea (26.4 % of children in the previous 2 weeks) [13]. Although very few studies have examined sanitation in Afghanistan specifically, the role of hygienic practices, potable water and sanitation in prevention of diarrhea is well established [15, 19–21]. Aggregate results from five studies have shown the relative risk (RR) of diarrhea to be 0.56 for handwashing interventions, and, in another analysis pooling results from 12 studies, the RR of diarrhea for household treatment of water was 0.65 [19]. Improved water supply and sanitation measures not only impact diarrhea and other diseases with fecal-oral transmission like helminth infections [20], but also pregnancy-related mortality [22].

### Policy and program recommendations

Multiple sanitary practices may need to be simultaneously implemented in underdeveloped areas given the numerous transmission routes for fecal contamination. According to the well-known F Diagram, disease is transmitted first from feces to fluids, fields, flies, or fingers, and then directly to a new host or indirectly through food [23]. Sanitation measures can effectively prevent transmission from feces, and water treatment and hand washing can prevent spread of disease from fluids and fingers, respectively. Because of limited literacy and governmental dysfunction in Afghanistan [21], there is difficulty in implementing interventions, both for educational interventions directed at individuals (from the government or non-governmental organizations (NGO)) and for community-wide improvements funded by the government or an NGO.

Hygiene and sanitation education at the individual or household level needs to be clear and simple and should include the following five key points; 1) Wash hands with soap, 2) Use the most protected water source available for drinking water, 3) Maintain latrines properly, 4) Protect food from contamination and 5) Use ORS when diarrhea occurs. However, the effectiveness of educational interventions in developing countries show mixed results; for example, telling or teaching individuals to wash their hands does not always translate to long-term behavioral changes [24, 25]. One study aiming to increase sanitation and health behaviors to decrease diarrheal disease in rural Afghanistan found limited effectiveness from educational interventions and concluded that it is better to promote integrated interventions, like the provision of water treatment chemicals, training in the use of water storage containers, and hygiene education, than any one intervention by itself [21]. The high prevalence of illiteracy in Afghanistan also limits the usefulness of educational campaigns that rely largely on written materials. More research is needed as to the optimal strategies for positively impacting health behaviors in underdeveloped settings.

From the governmental perspective, provision of safe, piped water into households and construction of more household latrines with a flush mechanism for residents of the underdeveloped areas are potential long-term solutions to reduce diarrheal risk. However, given the lack of resources in Afghanistan this could take years to successfully implement and simpler interventions could be implemented in the interim. Basic chlorine treatment of the water at the community level, filtration using simple household filters, and boiling could be promoted, subsidized, or even given to households. These interventions may have as great an impact on the health of the households as access to safe drinking water and basic sanitation services for populations at risk in underdeveloped area. However, such projects in Afghanistan are not only subject to limited government finances, but many underdeveloped areas, including the slum and IDP camp, have ambiguous legal status, so that municipal authorities are not responsible for their development. For example, the slum in this study was built on private land, and the IDP camp spontaneously developed on government-owned land that had not been developed. A clearer delineation of roles and delegation of authority would conceivably benefit municipal and national government officials who are charged with improving the living conditions of the thousands of families who live in Afghanistan’s underdeveloped areas. Educational campaigns to encourage hygienic behaviors such as handwashing can best effect behavioral change when infrastructure, such as piped water, is available to residents.

Finally, it is important to note the important role of women in any efforts to implement preventive interventions for diarrhea or other diseases in Afghanistan. Because we asked about household- and not individual-level prevalence of diarrhea, we did not focus on sex differences, but in most underdeveloped areas of Afghanistan, women and children are the primary water collectors and the female head of household exercises control over drinking water once it is collected. Therefore, short-term projects targeting sanitation education for women, and longer-term projects to improve female education generally could have a disproportionately positive impact upon the burden of diarrheal illness in the country although this is especially difficult given large numbers of displaced women within the country [5].

### Strengths and limitations

This study has a number of limitations. We acknowledge that a 3-month recall of diarrhea is subject to bias, and that a shorter duration such as 7 days leads to more valid results [26]; our choice of 3 months was based on our desire to estimate occurrence of disease throughout an entire season. Because this study was cross-sectional, it is possible adherence to certain sanitary behaviors increased following an episode of diarrhea in the household, resulting in reverse causation bias in our results. The study had a limited sample size and consisted of only two clusters. The lack of association between preventive behaviors and diarrhea in the household could be due to limited power. Additionally, the higher prevalence of diarrhea in the household in the IDP camp than the slum may be due to several variables intrinsic to the locations which were not evaluated, including density of dwellings, nutrition status of residents, and prevalence of bacteria and helminths in the environment. Other factors, like nutrition [13], also have a potentially enormous impact on disease states but were not assessed in this study. The two settings studied may not be representative of all IDP camps or all slums in Afghanistan although they do highlight a shortage of adequate sanitation measures in both locales. Despite these limitations, it is important to conduct and disseminate public health research on difficult to reach populations such as those described here. A number of studies have been published on disease burden among American military forces in Afghanistan [27, 28], but little is known about the prevalence of disease in Afghan people themselves. Because cultural perceptions of diarrhea may differ between native Afghans and US troops and given the very different demographic profile of these populations, any comparisons between the two, including for diarrheal illness, may not be valid.

A strength of our study was very high participation rates in both settings. This high rate resulted from a combination of study participants expressed interest in improving their circumstances and a commonly held belief that participating in the survey would disseminate knowledge about the difficult conditions in which they live.

## Conclusions

We found high prevalence of diarrhea in the household and infrequent use of sanitation and hygienic practices in two high-risk settings in Kabul, Afghanistan: an IPD camp and a slum. Because of the difficulties in implementing research in areas of extreme poverty located in regions of civil or military strife, few studies have examined the burden of disease in the Afghanistan as a whole, and fewer have looked specifically at the situation in high-risk settings. Interventions to improve sanitation, to increase access to potable water, and to encourage use of more hygienic behaviors can be undertaken and evaluated in order to reduce the substantial burden of diarrheal illnesses. Effective diarrhea control will not only require a change in behaviors and practices of persons who are largely illiterate and who reside in poor living conditions, but it will also require increased attention from NGO and government public health practitioners.

## Additional file

Additional file 1: Supplemental materials. (PDF 958 kb)

## Abbreviations

CI: confidence interval
HDI: human development index
IDP: internally-displaced person
KAP: knowledge, attitudes, and practice
NGO: non-governmental organization
OR: odds ratio
ORS: oral rehydration solution
RR: relative risk
