# Supplementary material for: Hygienic practices and diarrheal illness among persons living in at-risk settings in Kabul, Afghanistan: a cross-sectional study
Source: BMC Infect Dis. 2016 Aug 31;16(1):459. doi: 10.1186/s12879-016-1789-3 (PMC5006562; doi:10.1186/s12879-016-1789-3)
Supplement: Additional file 1: — Supplemental materials. (PDF 958 kb) [file 12879_2016_1789_MOESM1_ESM.pdf]

Supplemental Materials

English questionnaire

Dari questionnaire

DRINKING WATER SAFTETY AND SANITATION IN RELATION TO DIARRHEAL DISEASES IN UNDER DEVELOPED AREAS OF DISTRICT 5, KABUL

Questionnaire

ID Number: ☐

Date: ☐☐☐☐☐☐☐☐

DD/MM/YY

Was the Interview completed? Y ☐ N ☐

Informed Consent obtained (tick) ☐ Drinking water sample obtained (tick) ☐

Age of respondent (In completed years):

Number of household members in total:_______

Sex: 1 -male ☐ 2-female ☐

Number of under 5 children in total :_______

Section I: General Information (GI)			
**No**	**Questions**	**Answers**	**Skip**
1	What is the education level of the head of the household?	Illiterate……………………………………1<br>Below Primary…………………………..2<br>Primary …………………………………3<br>Middle …………………………………..4<br>Higher Education………………………..5	
2	How long has your family been living in this house? (Probe with available answers).	Less than one year... .... ………….…..... 1<br>One year to three years.....………………........ 2<br>More than three years... .....………………..... 3	

Section II: Drinking Water and Hygiene Practices of the Household			
**No.**	**Questions**	**Answers**	**Skip**
3	What is the principal source of drinking water for members of your household?<br>(CIRCLE ONE)	Dug wells (Protected)………………………1<br>Dug wells (Un- Protected)…………………..2<br>Hand pump(Protected)..................................3<br>Hand pump(Un-Protected) ………………..4<br>Tube well or borehole...................................5<br>Traditional Karez..6<br>Pipe water...7<br>Others ....8<br>Specify ______________________	
4	Where is your principal source of drinking water Located?	In dwelling. .... 1<br>In yard/compound ... .2<br>Outside yard/plot/, shared private source........3<br>Outside yard/plot/, shared public source..........4<br>Don’t know...8	
5	How long does it take to get the drinking water? *(by walk)*	Minutes ___ ___	
6	How do the members of the household usually wash their hands before taking meals?	warm water with soap ....…1<br>cold water with soap ....…2<br>warm water without soap ....…3<br>cold water without soap.............................…4<br>Don’t wash …………………………………5<br>Don’t know...8	
7	How do the members of the household usually wash their hands after defecation (latrine use)?	warm water with soap .... …1<br>cold water with soap ....…2<br>warm water without soap ....…3<br>cold water without soap ....…4<br>Don’t wash ………………………………...5<br>Don’t know 8	
8	Is it necessary for each person of household to wash hands every time when it gets dirty?	Yes .... ….1<br>No.. ….2<br>Not sure ....…8	If yes→ to Q10
9	If not, what is the reason?	Water not available ....….1<br>Soap not available ....….2<br>No need to wash …………………………...3<br>Don’t know ....….8	
10	Can I see your drinking water source?	Observed Yes ……………………………..1<br>Observed No ……………………………..…2<br>Not Allowed to Observe …………………....3	

11	If respondent allows, Is there flies seen around those places?	Yes ....1<br>No..2	
12	Where do you dispose of solid waste of household?	open..1<br>truck ....2<br>Other ....3<br>Specify__________________	

Section III: Knowledge and Practice about Water Sanitation & Hygiene

No.	Questions	Answers	Skip
13	Do you have separate container for storing drinking water?	Yes...1<br>No..2<br>Not applicable 3	If no → to Q15
14	Does this water container have a cover?	Yes ....1<br>No..2	
15	For how long do you usually store drinking water at home?	Less than one day ....1<br>One day..2<br>Two days ....3<br>From three days to one week.........................4<br>More than one week 5	
16	Do you have a place where you usually wash your hands, and if so, where is it, can I see it?	Yes, inside or next to sanitation facility ....1<br>Yes, inside or next to kitchen..........................2<br>Yes, inside living quarters ....3<br>Yes, outside in yard ....4<br>No..5	
17	Usually, do you treat water before drinking in order to make it safe?	Yes...1<br>No ....2<br>Don't know...3	If not or dk →19
18	If yes, which treatment do you apply to make it safer for drinking?<br><br>(circle all that apply)	Boil. ....1<br>Bleach/chlorine...2<br>Filter it through cloth.......................... 3<br>Water filter (ceramic, sand, composite) ....4<br>Solar disinfection..5<br>Salt..6<br>Others ....7<br>Specify ____________________	
19	Is it necessary to boil water every time before drinking?	Yes ....1<br>No..2<br>Not sure 8	

Section IV: Diarrheal diseases and Water Contamination

No.	Questions	Answers	Skip
20	Did any of your household members including your children under the age of 5 years suffer from Diarrhea during last three months? (Try to probe and find the answer)	Yes..1<br>No...2<br>Not sure..8	If no → to Q23
21	Had you or any other member of your household seen during passing stool?	Blood...1<br>Mucous ....2<br>Normal stool 3	

		Worms …… ………………………………….4<br>Other……………………………………….5<br>*Specify* _______________________	
22	During the episode of diarrhea, was your child given to drink a fluid made from special Packets called ORS?<br>ORS= Oral Rehydration Salts	Yes………………………………………..1<br>No…………………………………………2<br>Not sure…………………………………..8	If no → to Q24
23	If yes, Which type of ORS did you give to your child?	ORS pockets………..……………………..1<br>ORS solution………………………………2<br>Home-made fluid……………..…………...3	
24	Do you know, what can cause Diarrhea?<br>(circle all that apply)	Eating with dirty and unwashed hands ..……1<br>Dirty drinking water ....…2<br>Microbes ....…3<br>Insufficient hygiene ....…4<br>Don’t know ....…8	
25	Have you heard of Dehydration?	Yes………………………………………...1<br>No…………………………………………..2	
26	If any member of your household (including children) has diarrhea, will you consult a doctor?	Yes………………………………………….1<br>No…………………………………………..2<br>Not sure…………………………………….8	If no → to Q28
27	If No, what are the reasons of not consulting a doctor?	No Access to the Doctor ……………..…...1<br>Don’t have resources ……………..…..…..2<br>Others (Specify)_____________________ 3	
28	Do you think that diarrhea/dysentery can kill a person?	Yes ....…1<br>No ....…2<br>Not sure .... …8	
29	Have any of your family members including elders and under five years old children suffered from the following diseases?<br>(circle all that apply)	Dysentery…………………………………..1<br>Amoebiasis ………………………………..2<br>Giardiasis ………………………………….3<br>Typhoid……………………………………...4<br>Cholera... …………………………………..5<br>Worm manifestation…………… ..…………6<br>Other……………………………………….7<br>*Specify* _______________________	
30	How safe is running water?	It is always safe ....…1<br>It is not safe and clean ....…2<br>we don’t use running water......................…3<br>Don’t know ....…8	

Section V: Waste disposal and Sanitation Facilities

No.	Questions	Answers and Coding Categories	Skip
31	Does your household have a sanitation facility (latrine)?	Yes..1<br>No ....…2	If No→43
32	If yes, what type of latrine do you have?<br>Observation	Vault ....…1<br>Elevated Vault ....…2<br>Pit (>1 Meter deep)................................…3<br>Pour-flush..…4<br>Soak Pit ....…5<br>Other………………………………………....8<br>*Specify* _______________________	
33	Where is the latrine located?<br>May I see it please?	In compound yard...................................…1<br>Inside house..…2<br>Outside compound..................................…3<br>Other (Specify) ____________________4	
34	Is semi-solid or liquid waste draining from latrine on any side? Observation	Yes..1<br>No…………………………………….....…2	

35	Does the latrine have a separate urine pipe? *(Please observe and record the appropriate answer)*	Yes, goes into ground ....1<br>Yes, open at back of latrine...........................2<br>Yes, but broken..3<br>No 4	
36	Does the latrine have a ventilation pipe? *(Please observe and record the appropriate answer)*	Yes..1<br>No 2	
37	Distance from latrine to drinking water Source?<br><br>*(Please observe and record the appropriate answer)*	Less than 5m...1<br>5-10m ....2<br>10-15m...3<br>15-20 m...4<br>20-30m...5<br>Above 30m 6	
38	Availability of flies inside or outside latrine?	Yes..1<br>No 2	
39	How often is the latrine is emptied (dislodged)?	Every Year ....1<br>Every 6 months ....2<br>Every 3 months ....3<br>Every month ....4<br>Every week ....5<br>Never ....6<br>Not Applicable ……………………………..7	
40	Do you disinfect the pit latrine and soak pit?	Yes..1<br>No 2	If no → to Q43
41	If [yes] how often.	Every month ....1<br>Every week ....2<br>Every day ....3<br>Never ....4<br>Other……………………………………...…8<br>*Specify* ________________________	
42	What do you use to disinfect the pit latrine and soak pit?	ash..1<br>salt ....2<br>chlorine...3<br>Other...4<br>Specify___________________	
43	How many households **including you** share the latrine?	One household..1<br>Two household ....2<br>Three household or more............................3<br>Don't know...8	

That is the end of the questionnaire. Thank you very much for your valuable time and cooperation. If you would like to talk about water and sanitation with me, we can do that now.

How does the interviewer rate the validity of information given	Good……………………………………………..1<br>Average……………………………………….2<br>Poor…………………………………………….3
How cooperative was the respondent?<br>1 = Very much cooperative, 2= cooperative, 3= Not cooperative	1 2 3
How interested was the respondent in the survey?<br>1= Very much interested, 2= Interested; 3= Not interested	1 2 3

حفظ الصحه و امراض اسهالی در بین باشنده گان منطقه در معرض خطر شهر کابل، افغانستان:

مطالعه تحقیقی مشاهداتی مقطعی

سوالنامه

شماره مسلسل ☐

تاریخ:

روز ماه سال

آیا مصاحبه تکمیل گردیده است؟ بلی ☐ نخیر ☐

رضایت اگاهانه گرفته شده است (نشانی گردد) ☐ نمونه آب گرفته شده است (نشانی گردد) ☐

سن و سال پاسخ دهنده (به سال مکمل)

تعداد مجوعی اعضای خانوار:________

جنس: 1- ذکور ☐ 2 -اناث ☐

تعداد مجموعی اطفال زیرپنج سال: ________

بخش اول (معلومات عمومی)			
**شماره**	**سوالات**	**جوابات**	**مراجعه به بخش بعدی**
1	رئیس فامیل کدام سطح تعلیمی دارد؟	بیسواد.........1<br>ازابتدائیه پایین.........2<br>ابتدائیه ....3<br>متوسطه ....4<br>تحصیلات عالی 5	
2	برای چه مدت زمان فامیل شما در این خانه زندگی میکند؟<br>(از میان جوابات موجود انتخاب نمایید	کمتر از یکسال......... 1<br>از یک تا سه سال......... 2<br>اضافه تر از سه سال......... 3	

بخش دوم: عادات حفظ الصحه آب در خانوار			
**شماره**	**سوالات**	**جوابات**	**مراجعه به بخش بعدی**
3	منبع اصلی آب آشامیدنی اعضای فامیل شما چه است؟<br>(تنها یک جواب را انتخاب نمایید	چاه (محفوظ).........1<br>چاه (غیر محفوظ).........2<br>بمبه دستی (محفوظ) ....3<br>بمبه دستی (غیرمحفوظ) ....4<br>چاه عمیق ....5<br>آب کاریز.........6<br>آب نل ....7<br>سایر.........8<br>مشخص نمایید ________	
4	منبع اصلی آب آشامیدنی در کجا واقع شده است؟	در خانه.........1<br>در حویلی ....2<br>خارج از حویلی در ساحه شخصی شریک.........3<br>خارج از حویلی در ساحه عامه شریک.........4<br>نمیفهمم.........8	
5	برای بدست آوردن آب آشامیدنی چه مدت زمان به کار است؟<br>*(پیاده)*	دقایق ___ ___	
6	اعضای فامیل شما معمولا قبل از گرفتن مواد غذایی چه قسم دست های خود را میشویند؟	با آب گرم همراه صابون.........1<br>با آب سرد همراه صابون.........2<br>با آب گرم بدون صابون ....3<br>با آب یخ بدون صابون.........4<br>نمی شوییم ....5<br>نمی فهمم 8	
7	اعضای فامیل شما معمولا بعد از استفاده از بیت الخلا چه قسم دست های خود را میشویند؟	با آب گرم همراه صابون.........1<br>با آب سرد همراه صابون.........2<br>با آب گرم بدون صابون.........3<br>با آب یخ بدون صابون.........4<br>نمی شوییم ....5<br>نمی فهمیم 8	

8	آیا نیاز است تا هر کدام از اعضای خانوار دست های خود را هر بار بعد از ملوث شدن بشویند؟	بلی..1<br>نخیر..2<br>مطمئن نیستم..8	در صورتی که جواب بلی باشد به جواب 10↓ مراجعه نمایید
9	در صورتیکه جواب نخیر باشد، علت آن چیست؟	آب موجود نیست...1<br>صابون موجود نیست ....2<br>ضرورت به شستن نیست ....3<br>نمی فهمم 8	
10	آیا میتوانم منبع آب آشامیدنی شما را ببینم؟	بلی مشاهده شد ....1<br>نخیر مشاهده نشد ....2<br>اجازه داده نشد تا مشاهده گردد...........................3	
11	در صورتیکه پاسخ دهنده اجازه دهد، آیا مگس ها در اطراف محلات دیده میشود؟	بلی..1<br>نخیر..2	
12	شما فضله جات جامد را در کجا جابجا مینمایید؟	باز..1<br>بیرل کثافات و فضله جات ....2<br>سایر...3<br>مشخص نمایید ____________________	

بخش سوم: دانش و مراعات حفظ الصحه و آب

شماره	سوالات	جوابات	مراجعه به بخش بعدی
13	آیا شما جهت ذخیره نمودن و نگهداری آب ظرف جداگانه دارید؟	بلی..1<br>نخیر..2<br>موجود نیست 3	در صورتی که جواب نخیر باشد به سوال 15 مراجعه نمایید↓
14	آیا ظرف آب شما سرپوش دارد؟	بلی..1<br>نخیر..2	
15	برای چه مدت زمان شما آب را در خانه ذخیره و نگهداری میکنید؟	کمتر از یک روز..1<br>یک روز..2<br>دو روز...3<br>از سه روز تا یک هفته....................................4<br>اضافه تر از یک هفته 5	
16	آیا شما کدام جای برای شستن دست ها دارید، اگر موجود است، در کجا است، میتوانم آن را ببینم؟	بلی در داخل است، نزدیک تشناب.........................1<br>بلی در داخل آشپزخانه.....................................2<br>بلی در داخل محل بودوباش...............................3<br>بلی، بیرون از حویلی ....4<br>نخیر 5	

17	معمولا شما آب را قبل از نوشیدن جهت پاک شدن ضدعفونی مینمایید؟	بلی .... 1<br>نخیر........ 2<br>نمی فهمم 3	در صورتی که جواب نخیر باشد به سوال 19 مراجعه نمایید↓
18	در صورتیکه جواب بلی باشد، به کدام شکل آن را ضد عفونی مینمایید؟<br>(تمام جوابات داده شده را نشانی کنید)	جوش دادن........ ....1<br>استفاده از بلیچ/ کلورین........ ...2<br>فلتر نمودن از تکه........ 3<br>فلتر آب ساخته شده از (سیرامیک، ریگ) ....4<br>ضد عفونی نمودن توسط سولر........ ...5<br>نمک ....6<br>سایر........ ...7<br>مشخص نمایید ____________	
19	آیا نیاز است تا قبل از نوشیدن آب جوشانده شود؟	بلی........ ...1<br>نخیر........ ...2<br>مطمئن نیستم........ ...8	

بخش چهارم: امراض اسهالی و ملوثیت آب			
**شماره**	**سوالات**	**جوابات**	**مراجعه به بخش بعدی**
20	آیا کدام عضو فامیل شما به شمول اطفال کمتر از پنج سال در جریان سه ماه گذشته مصاب اسهال شده اند (کوشش شود تا جواب دریافت گردد)	بلی........1<br>نخیر........2<br>مطمئین نیستم........8	در صورتیکه جواب نخیر باشد به سوال 23 مراجعه نمایید↓
21	آیا شما یا کدام عضو دیگر فامیل تان یکی از ذیل را در مواد غایطه خود دیده اید؟	خون........1<br>مخاط ....2<br>مواد غایطه نورمال ....3<br>کرمها ....4<br>سایر........5<br>مشخص نمایید____________	
22	آیا به طفل تان مایع ساخته شده از پودر(او آر اس) در هنگام اسهال داده اید؟	بلی........1<br>نخیر........2<br>مطمئن نیستم........8	در صورتیکه جواب نخیر باشد به سوال 24 مراجعه نمایید↓
23	درصورتیکه جواب بلی باشد، کدام قسم او آر اس را به طفل خود میدهید؟	پاکت های او آر اس........1<br>محلول او آر اس........2<br>مایعات تهیه شده در منزل........3	
24		خوردن با دست های شسته نا شده و ملوث 1	

	آیا شما میفهمید که چه باعث اسهال میشود؟<br>(تمام جوابات ارائه شده را حلقه نمایید)	آب ملوث ....2<br>مایکروبها ....3<br>حفظ الصحه ناکافی ....4<br>نمیفهمم<br>...8	
25	آیا شما در مورد دیهایدریشن شنیده اید؟	بلی...1<br>نخیر...2	
26	در صورتیکه کدام عضو فامیل شما (به شمول اطفال) مصاب اسهال باشند، آیا شما به داکتر مراجعه مینمایید؟	بلی...1<br>نخیر..2<br>مطمین نیستم.................................8	در صورتیکه جواب نخیر باشد به سوال 28 مراجعه نمایید↓
27	در صورتیکه جواب نخیر باشد، عامل عدم مراجعه شما به داکتر چیست؟	به داکتر دسترسی نداریم ....1<br>منابع نداریم ....2<br>سایر (مشخص نمائید)......................................3	
28	آیا شما فکر میکنید که اسهال/ پیچش شخص را کشته میتواند؟	بلی ....1<br>نخیر..2<br>مطمئن نیستم 8	
29	آیا کدام عضو فامیل شما به شمول اطفال زیر پنج سال و بزرگسالان مصاب یکی از امراض ذیل شده اند؟<br><br>(تمام جوابات داده شده نشانی گردد)	پیچش...1<br>امیبیازس ....2<br>جیاردیازس ....3<br>محرقه...4<br>کولرا... ....5<br>مصابیت به کرمها............... ....6<br>سایر..7<br>مشخص نمایید ____________________	
30	به نظر شما آب جاری به چه اندازه مصئون است؟	همیشه مصئون است ....1<br>مصئون و پاک نمیباشد ....2<br>ما از آب جاری استفاده نمیکنیم ....3<br>نمیفهمم..8	
**بخش پنجم: جابجانمودن فضله جات و تسهیلات حفظ الصحوی**			
**شماره**	**سوالات**	**جوابات و بخش های کود گذاری**	**مراجعه به بخش بعدی**
31	آیا فامیل شما بیت الخلا (تشناب) دارید؟	بلی ....1<br>نخیر..2	در صورتیکه جواب نخیر باشد به سوال 43 مراجعه نمایید↓
32	در صورتیکه جواب بلی باشد، کدام نوع بیت الخلا دارید؟ مشاهده شود.	سوراخ چقر...1<br>سوراخ چقر وسیع ....2<br>خندق به عمق زیادتر از 1 متر ....3<br>بیت الخلای با ریختن آب توسط فلش........................4<br>بیت الخلا با ریختن آب...5<br>سایر...8<br>مشخص نمایید ____________________	
33	بیت الخلای شما در کجا واقع شده است؟ میتوانم آن را ببینم؟	در بین حویلی ....1<br>در بین منزل..2<br>خارج از خانه ....3<br>سایر (مشخص نمایید)____________________4	

34	آیا فضله جات و غایطه نیمه جامد یا مایع در اطراف بیت الخلا جریان دارد ؟ مشاهده شود.	بلی ....1<br>نخیر...2	
35	آیا بیت الخلا نل جداگانه دارید؟<br>*(لطفا مشاهده نمایید و جواب مناسب را انتخاب نمایید)*	بلی در بین زمین میرود..........................1<br>بلی به عقب بیت الخلا باز است..........................2<br>بلی اما شکسته است..........................3<br>نخیر 4	
36	آیا بیت الخلا نل تهویه دارد؟ (لطفا مشاهده نمایید و جواب مناسب را انتخاب نمایید)	بلی ....1<br>نخیر...2	
37	فاصله میان بیت الخلا و منبع آب آشامیدنی؟<br>*(لطفا آن را مشاهده کنید و جواب مناسب را انتخاب کنید)*	کمتر از 5 متر...1<br>5-10 متر..2<br>10-15 متر..3<br>15-20 متر..4<br>20-30 متر..5<br>اضافه تر از 30 متر......................................6	
38	آیا مگس ها در داخل یا خارج بیت الخلا موجود اند؟	بلی...1<br>نخیر...2	
39	به چه فاصله زمانی بیت الخلای تان را تخلیه میکنید؟	سالانه...1<br>هر شش ماه ....2<br>هر سه ماه ....3<br>ماهانه ....4<br>هفته وار ....5<br>هیچگاه ....6<br>موجود نیست 7	
40	آیا شما در بیت الخلای تان از مواد ضد عفونی استفاده میکنید؟	بلی ....1<br>نخیر..2	در صورتیکه جواب نخیر باشد به سوال 43 مراجعه نمایید ↓
41	در صورتیکه جواب بلی باشد، در جریان چه مدت زمان؟	ماهانه ....1<br>هفته وار ....2<br>روزانه ....3<br>هیچگاه ....4<br>سایر..8<br>مشخص نمایید ___________________________	
42	جهت ضد عفونی نمودن بیت الخلای تان شما از کدام مواد استفاده مینمایید؟	خاکستر ....1<br>نمک...2<br>کلورین..3<br>سایر ....4<br>مشخص نمایید___________________	
43	به شمول شما چند خانوار از بیت الخلای موجود استفاده مینمایند؟	یک خانوار ....1<br>دو خانوار ....2<br>سه یا اضافه تر از سه خانوار....................................3<br>نمی فهمم 8	

سوالنامه ما خاتمه یافت. تشکر از وقت گرانبها و همکاری شما. در صورتیکه شما بخواهید در مورد حفظ الصحه آب صحبت کنید، میتوانیم حال صحبت نماییم.

مصاحبه کننده اعتبار معلومات داده شده را چگونه نمره دهی مینماید.	خوب...1<br>متوسط...2<br>خراب...3
پاسخ دهنده تا چه اندازه در هنگام مصاحبه همکار بود؟	1 2 3

1= بسیار زیاد همکار، 2= همکار، 3= عدم همکاری	
پاسخ دهنده در مورد سروی تا چه اندازه علاقه مند بود؟<br>1= بسیار زیاد علاقمند، 2= علاقمند، 3= عدم موجودیت علاقه	1 2 3
